# Atrial Fibrillation and Coronary Artery Disease as Risk Factors of Retinal Artery Occlusion: A Nationwide Population-Based Study

**DOI:** 10.1155/2015/374616

**Published:** 2015-10-19

**Authors:** Ju-Chuan Yen, Hsiu-Li Lin, Chia-An Hsu, Yu-Chuan (Jack) Li, Min-Huei Hsu

**Affiliations:** ^1^Graduate Institute of Biomedical Informatics, College of Medical Science and Technology, Taipei Medical University, Taipei, Taiwan; ^2^Department of Ophthalmology, Taipei City Hospital, Zhongxiao Branch, Taipei 115, Taiwan; ^3^Department of Neurology, Cathay General Hospital, Sijhih Branch, Taipei, Taiwan; ^4^School of Medicine, National Yang-Ming University, Taipei 115, Taiwan; ^5^Ministry of Health and Welfare, No. 488, Sec. 6, Zhongxiao E. Road, Taipei 115, Taiwan

## Abstract

We use Taiwanese national health insurance research database (NHIRD) to investigate whether thrombolism (carotid artery disease (CAD) as a surrogate) or embolism (atrial fibrillation (AF) as a surrogate) plays roles in later retinal artery occlusion (RAO) development and examine their relative weights. The relative risks of RAO between AF and CAD patients and controls were compared by estimating the crude hazard ratio with logistic regression. Kaplan-Meier analysis was used to calculate the cumulative incidence rates of developing RAO, and a log-rank test was used to analyze the differences between the survival curves. Separate Cox proportional hazard regressions were done to compute the RAO-free rate after adjusting for possible confounding factors such as age and sex. The crude hazard ratios were 7.98 for the AF group and 5.27 for the CAD group, and the adjusted hazard ratios were 8.32 and 5.34 for the AF and CAD groups, respectively. The observation time with RAO-free was shorter for AF compared with CAD group (1490 versus 1819 days). AF and CAD were both risk factors for RAO with different hazard ratios. To tackle both AF and CAD is crucial for curbing RAO.

## 1. Introduction

Central retinal artery occlusion was first described by Dr. Von Graefe in 1859 [1–3]. The extent to which thrombosis or embolism plays a role in causing retinal artery occlusion is a perennial question of ophthalmologists. Hayreh proposed that the etiology of emboli is the most common cause of retinal artery occlusion [2, 4]. We used the national health insurance database in Taiwan to delve into the possible linkage of etiologies for retinal artery occlusion to understand whether embolism or thrombosis plays a role in causing retinal artery occlusion or both of them play a role. In this study, atrial fibrillation was used as a surrogate of embolism and coronary artery disease as a representative of thrombosis. Based on this hypothesis, there then should be statistically significant results for the atrial fibrillation and coronary artery disease study group patients to develop retinal artery occlusion later. And if embolism is the major cause of RAO, then the hazard ratio for atrial fibrillation patients to develop RAO would be higher than coronary artery disease patients and vice versa.

In Taiwan, the government launched the national health insurance as a mandate on 1 March 1995; and the coverage rate is around 99% [5]. A nationwide population study using a longitudinal case-controlled cohort study was conducted to examine whether atrial fibrillation and/or coronary artery disease was or were risk factor(s) for retinal artery occlusion and to compare the relative hazard ratios between these two groups of patients. The Longitudinal Health Insurance Database 2000 (LHID2000) is a subdataset of the national health insurance research database (NHIRD), which includes all claims data (from 1996 to 2008) of one million beneficiaries who were randomly selected from the system in 2000. There was no significant difference in age, sex, or average insured payroll-related premiums between the sample group and all enrollees.

## 2. Materials and Methods

### 2.1. Selection of Patients and Variables

This cohort comparison study consisted of all patients diagnosed with atrial fibrillation (AF) (*n* = 9,756) (ICD-9-CM codes 427.31) and coronary artery disease (CAD) (*n* = 95421) (ICD-9-CM codes 410, 411, 412, 413, and 414) from ambulatory (including emergency) care and inpatient care, from 1 January 2000 through 31 December 2008 in the LHID 2000. The control patients are four patients for every atrial fibrillation (AF) patient and one patient for each coronary artery disease (CAD) patient as patients not diagnosed with AF or CAD, which were randomly selected from the dataset. The reason the coronary artery disease cohort group could only be a one to one ratio is that the sample size of the CAD study group was too large, so we could not obtain enough control patients to be greater than the rate of 1 : 1. The patients included in the study and control group patients were matched by sex, age, and the index date of ambulatory care visit (including outpatient clinic and emergency department) or hospitalization for the initial diagnosis of AF or CAD patients. And among these datasets, we further examined all patients who had ever developed retinal artery occlusion after a diagnosis of atrial fibrillation or coronary artery disease as RAO (ICD-9-CM codes 362. 31 (central retinal artery occlusion (CRAO)), 362.32 (branch retinal artery occlusion (BRAO)), and 362.33 (partial retinal artery occlusion (PRAO)). Demographic data, such as sex and age, were recorded.

### 2.2. Statistical Analysis

*SAS for Windows 9.3* (SAS Institute, Inc., Cary, North Carolina, USA) was used for this study. Descriptive statistical analyses were done to compare the characteristics of the two cohorts in terms of demographic characteristics and the risk of developing retinal artery occlusion. The risk of retinal artery occlusion between AF and CAD patients and controls was compared by estimating the crude hazard ratio with logistic regression. Logistic regression is widely used in analysis of categorical data especially data with variables that have binary responses. It can predict a dichotomous outcome using independent variables. This dichotomous outcome is presence or absence of RAO in this study. Kaplan-Meier analysis was used to calculate the cumulative incidence rates of developing retinal artery occlusion between the two different cohorts, and the log-rank test was used to analyze the differences between the survival curves. Thereafter, separate Cox proportional hazard regressions were done to compute the RAO-free rate after adjusting for possible confounding factors such as age and sex. Cox regression is method for investigating the effect of variables upon the time a specified event takes to happen. The coefficients in a Cox regression relate to hazard; a positive coefficient indicates a worse prognosis and a negative coefficient indicates a protective effect of the variable with which it is associated. Statistical significance was set at *p* ≤ 0.05.

## 3. Results

### 3.1. Demographic Data

Between 2000 and 2008, 9,756 AF patients and 38,872 AF control patients and 95421 CAD patients and 95419 CAD control patients, with age-, sex-matched controls, were recruited, after excluding ineligible subjects. The median age of the AF patients was 68.9 years old and for the controls it was 68.3 years old. The median age of the CAD patients was 57.4 years old and for the controls it was 56.9 years old. The sex ratios between the two groups were as follows: M/F 54.6%/45.4% in the AF study group and 54.8%/45.2% in the AF control group; M/F 49.2/50.8 in the CAD study group and 49.1/50.9 in the CAD control group (Tables 1 and 2).

For the atrial fibrillation study group, there were 9,756 patients, and, among them, 18 patients (0.18%) developed retinal artery occlusion later, while, for the atrial fibrillation control group, there were 38,872 patients and 9 patients that (0.02%) developed retinal artery occlusion later. Therefore, there were 27 AF patients (0.05%) of 48,628 cases that developed RAO. On the other hand, for CAD study group, there were 95,421 patients and 79 cases that developed RAO later; and for CAD controls, there were 95,419 patients and, among them, 12 cases (0.02%) developed RAO later in the observation period. The differences in the risks of developing RAO in both groups were statistically significant (*p* < 0.0001). The median observation period and interquartile range between the two groups to develop the RAO were 1490 (666–1384) days in the AF study group, 1606 (728–1451) days in the AF control group, 1819 (1009–1882) days in the CAD study group, and 1854 (1016–1889) days for the CAD control group, respectively. The observation periods for the AF and CAD groups to develop RAO later differed in length. For the AF study group, it was around four years (4.08 years), while for the CAD study group it was around five years (5.07), meaning it will be around one year earlier to develop RAO later in AF patients than that of CAD patients. The differences in the survival analysis between the two groups, that is, the AF study group and AF control group and CAD study group and CAD control group, are statistically significant as well. The *p* value is <0.0001 (Tables 3 and 4).

**Figure 1:**
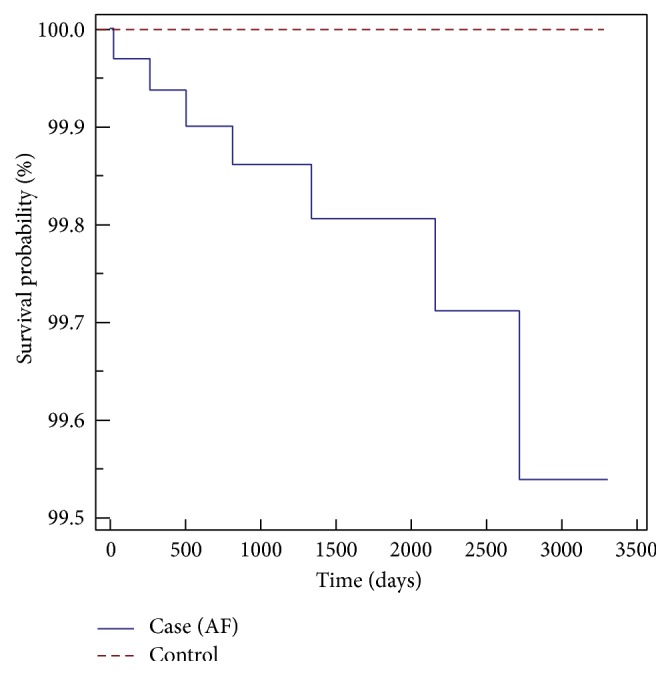
Kaplan-Meier survival analysis of AF patients with RAO-free time.

**Figure 2:**
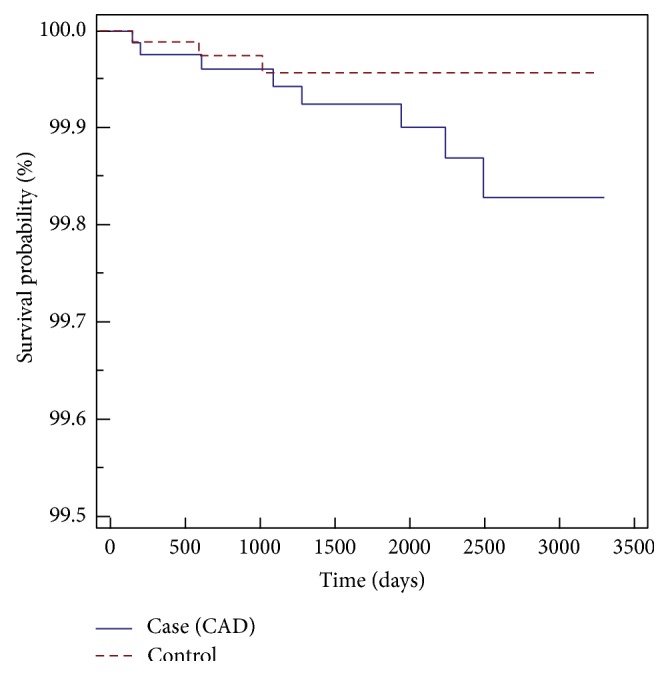
Kaplan-Meier survival analysis of CAD patients with RAO-free time.

Among the AF cohorts, the crude hazard ratio by logistic regression with a 95% confidence interval for the AF patients to develop RAO is 7.98 (3.59–17.77); and the adjusted hazard ratio by Cox proportional regression model is 8.32 (3.70–18.32). The adjusted factors included age and sex. For the CAD cohorts, the crude hazard ratio by logistic regression with 95% confidence interval for the CAD patients to develop RAO is 5.27 (3.03–9.15); and the adjusted hazard ratio by the Cox proportional regression model is 5.34 (3.27–9.26). The adjusted factors included age and sex. Kaplan-Meier survival analysis was conducted to examine the cumulative incidence rates of developing retinal artery occlusion between the two different cohorts, and a log-rank test was used to probe the differences between the survival curves. The results (Figures 1 and 2) revealed statistically significant differences between the two cohorts, that is, the AF cohorts and CAD cohorts, *p* < 0.0001. In AF and CAD cases, to develop RAO, the sex did not increase the hazard ratios, yet the hazard ratios increased 1.03 times for every 10 years of age in atrial fibrillation patients and 1.376 times for every 10 years of age in coronary artery disease patients.

## 4. Discussion

The results showed that atrial fibrillation (AF) and coronary artery disease (CAD) will both increase the risk of developing retinal artery occlusion (RAO) later, as the crude hazard ratios with 95% confidence interval were 7.98 (3.59–17.77) and 5.27 (3.03–9.15); and the adjusted hazard ratios with 95% confidence interval were 8.32 (3.70–18.32) and 5.34 (3..27–9.26) for AF patients and CAD cohorts, respectively. This means the hazard ratios, regardless of whether the crude or adjusted ones were adjusted for age and sex, are still both quite high for both AF and CAD group patients to develop RAO later; and the hazard ratio for the AF group is around 1.5 times higher than that of CAD group. This result seems to echo Hayreh's point of view that embolism is by far the most common etiology of RAO. Wong et al. suggested the etiology of RAO was from thrombus, which is a different point of view [6]. This reason that arteriosclerosis causes thrombus in the internal carotid artery or heart and further influences and/or simultaneously attacks the ocular vascular system such as the retinal arteries is plausible, as the hypertension and hyperlipidemia would cause artery wall atheroma formation or artery sclerosis, that is, resistance to changes in feasibility during stress and the alternating relaxing condition later on. In this way, the thrombus would form in the arterioles or arteries and result in the retinal artery occlusion event as the progression of atheroma plaques continues and the severity of arterial stenosis worsens.

We also found, between the two groups, it took one year less to develop RAO for the AF group (around four years) than that of the CAD group (around five years). In this respect, embolism raises our alertness to be a stronger promoting factor in RAO development. Therefore, for AF patients, as denoted by the cardiologist's perspective [7], one in four adults older than forty years old might develop AF later in their life, and many AF patients do not have conspicuous symptoms, so understanding the possible risk of developing RAO is critical because it might help prevent later visual tragedy and possible systemic misery. Clearly, detecting AF patients and subjecting them to anticoagulant treatment to prevent possible vascular events like the retinal artery occlusion are especially crucial for saving vision in this regard.

In clinical settings, ophthalmologists might not be convinced an embolism is by far the most common etiology for RAO because we could not see the emboli in the fundus in most RAO cases. However, as Hayreh proposed, this might be because the emboli in RAO were usually tiny and located in the microvascular bed. They could be dislodged later and therefore not show in the retinae. From our study, this viewpoint is also verified. For the RAO emboli sources, from the study of Arruga and Sanders in 1982, 74% were made of cholesterol, 10.5% of calcific material, and only 15.5% of platelet-fibrin. Some ophthalmologists have tried surgical embolectomy [8, 9] to treat RAO. In these studies, after removal of a calcified material embolism, some patients' visual acuity improved from no light perception to counting fingers. The reason some failed through surgical intervention might have been due to the delay in initiating treatment. As the clinical reality goes, most RAO patients do not visit ophthalmologists or physicians in the golden four hours. Most patients visit ophthalmologists several days later. Therefore, understanding the possible causes, appropriate management, and proper expectations are pivotal for both physicians and patients in preventing grave outcome of retinal artery occlusion events and reducing the disease burdens. Kirwan et al. revealed a case from Ireland where multiple amaurosis fugax attacks developed RAO later and with very poor visual acuity outcome as counting fingers was indeed a case of paroxysmal atrial fibrillation [10]. And so the authors highlighted the importance of Holter monitoring for the patients and in this way warfarin or other anticoagulant treatments are very critical in helping physicians including ophthalmologists and patients to deal with the disorders. A study conducted by Johns Hopkins Hospital [11] showed 42 CRAO patients between 1999 and 2006 with intra-arterial delivering of tPA in aliquots with the timeline up to 15 hours after attack of CRAO and found a statistically significant improvement in three lines of visual acuity or more of vision as compared with control subjects who did not receive thrombolysis. Yet the European Assessment Group for Lysis in the Eye (EAGLE) [12] conducted a multicentered, prospective randomized controlled trial of 84 patients with CRAO within 20 h of symptom onset and did not show a statistically significant difference in clinical improvement between the lysis and standard therapy groups (60.0 versus 57.1%). Thrombolysis can also be administered by other routes as intravenous tPA delivery. Better outcomes resulted from the starting treatment within 6.5 hours of CRAO attack in one case series [13]. There is no consensus on fibrinolytic thrombolysis treatment.

The cohort for AF was older (median age 68 years old) than that of CAD (median age 57 years old) in the Taiwanese population. Slight sex differences existed in the AF cohorts, with males in the AF group comprising 54.8%, while there is almost no difference in the CAD cohorts (F/M 49.8%/50.2%). There is no statistically significant risk difference in sex towards the later development of RAO, yet the increase of ten-year age rank would increase the risk 1.34-fold.

This study was conducted based on a single-payer longitudinal national health insurance database in Taiwan to probe the hazard ratios of AF and CAD as risk factors for retinal artery occlusion development and verify the hypothesis mentioned previously. Were it not for this NHIRD longitudinal dataset, it would be very difficult to collect enough cases to conduct the research because of the time-consumption and cost issues. And since it is a national health insurance database, it is a population-based study, so the results are more robust and convincing without selection bias.

On the other hand, the claims data of national insurance health database could not show important clinical features such as the retinal images and stereotactic fundus pictures of possible emboli and/or fluorescein angiography. It could also not show personal health records like the body mass index and exercise or food intake habits, which are important in interpreting the etiology of RAO.

And there are flaws in the claims data such as the fact that our inclusion criteria were based on ICD-9-CM codes, but there was no way to verify the accuracy of coding by physicians, and so there should be some expected defects in this respect by using national health insurance research database.

## 5. Conclusion

Taiwanese national health insurance database was used to examine the risk of atrial fibrillation and coronary artery disease in developing retinal artery occlusion later. The results revealed both AF and CAD will increase the risk of later developing RAO as the crude HRs with 95% confidence interval for both groups were 7.98 (3.59–17.77) and 5.27 (3.03–9.15), and the adjusted HRs with 95% confidence interval were 8.32 (3.70–18.32) and 5.34 (3.27–9.26). The embolism factor (surrogate as AF) is more important for the etiology of RAO than that of thrombolism (surrogate as CAD).

## Figures and Tables

**Table 1:** Demographics of AF group and control group.

Variable	AF patients (*n* = 9,756)		Control group (*n* = 38,872)		*p * value
	*n*	%	*n*	%	
Age, median (IQR^a^)	68.9 (10)		68.3 (10)		0.99
Gender					0.99
Male	5,324	54.6	21,288	54.8	
Female	4,432	45.4	17,584	45.2	
RAO	18	0.2	9	0.02	<0.0001^*∗∗∗*^
Observation time without developing RAO (days, median, IQR^a^)	1,490	(666–1384)	1,606	(728–1,451)	<0.001^*∗∗∗*^

^a^IQR: interquartile range, *∗* indicates *p* < 0.05, *∗∗* indicates *p* < 0.01, and *∗∗∗* indicated *p* < 0.001.

**Table 2:** Demographics of CAD group and control group.

Variable	CAD patients (*n* = 95,421)		Control group (*n* = 95,419)		*p* value
	*n*	%	*n*	%	
Age, median (IQR^a^)	57.4 (10)		56.9 (10)		0.99
Gender					0.99
Male	46,879	49.2	46,879	49.1	
Female	48,542	50.8	48,540	50.9	
RAO	79	0.08	12	0.02	<0.0001^*∗∗∗*^
Observation time without developing RAO (days, median, IQR^a^)	1819	(1009–1882)	1854	(1016–1889)	<0.001^*∗∗∗*^

^a^IQR: interquartile range and *∗∗∗* indicated *p* < 0.001.

**Table 3:** Crude and adjusted hazard ratios for developing retinal artery occlusion among patients with atrial fibrillation and the control group during the ten-year follow-up (*n* = 48,628).

Development of RAO	Total		Patients with AF		Control group	
	Number	%	Number	%	Number	%
Nine-year follow-up period						
Yes	27	0.05	18	0.18	9	0.02
No	48,601	99.95	9,738	99.82	38,863	99.98
Crude HR (95% CI)	—		7.98 (3.59–17.77)		1.00	
Adjusted^a^ HR (95% CI)	—		8.32 (3.70–18.32)		1.00	

^a^Adjustments were made for sex and age.

**Table 4:** Crude and adjusted hazard ratios for developing retinal artery occlusion among patients with coronary artery disease and the control group during the ten-year follow-up (*n* = 190,840).

Development of RAO	Total		Patients with CAD		Control group	
	Number	%	Number	%	Number	%
Ten-year follow-up period						
Yes	91	0.5	79	0.1	12	0.02
No	190,749	99.95	95,342	99.9	95,407	99.98
Crude HR (95% CI)	—		5.27 (3.03–9.15)		1.00	
Adjusted^a^ HR (95% CI)	—		5.34 (3.27–9.26)		1.00	

^a^Adjustments were made for sex and age.
